# Incidence, features, and prognosis of immune-related adverse events involving the thyroid gland induced by nivolumab

**DOI:** 10.1371/journal.pone.0216954

**Published:** 2019-05-14

**Authors:** Ichiro Yamauchi, Akihiro Yasoda, Shigemi Matsumoto, Yuichi Sakamori, Young Hak Kim, Motoo Nomura, Atsushi Otsuka, Toshinari Yamasaki, Ryoichi Saito, Morimasa Kitamura, Toshio Kitawaki, Masakatsu Hishizawa, Nobuko Kawaguchi-Sakita, Toshihito Fujii, Daisuke Taura, Masakatsu Sone, Nobuya Inagaki

**Affiliations:** 1 Department of Diabetes, Endocrinology and Nutrition, Kyoto University Graduate School of Medicine, Kyoto, Japan; 2 Department of Medical Oncology, Kyoto University Graduate School of Medicine, Kyoto, Japan; 3 Department of Respiratory Medicine, Kyoto University Graduate School of Medicine, Kyoto, Japan; 4 Department of Dermatology, Kyoto University Graduate School of Medicine, Kyoto, Japan; 5 Department of Urology, Kyoto University Graduate School of Medicine, Kyoto, Japan; 6 Department of Otolaryngology, Head and Neck Surgery, Kyoto University Graduate School of Medicine, Kyoto, Japan; 7 Department of Hematology and Oncology, Kyoto University Graduate School of Medicine, Kyoto, Japan; UT MD Anderson Cancer Center, UNITED STATES

## Abstract

**Background:**

Blocking the PD-1 pathway induces immune-related adverse events (irAEs) which often involve the thyroid gland (thyroid irAEs). Clinical features of a thyroid irAE including its predictability and relationship to prognosis remain to be elucidated.

**Methods:**

Two hundred consecutive patients treated with nivolumab at Kyoto University Hospital between September 1, 2014 and August 31, 2017 were included in a retrospective cohort study. We systematically determined and classified subclinical and overt thyroid irAEs based on data collected of serum free T4 and TSH levels. Baseline characteristics and detailed clinical data were analyzed, and analyses of overall survival (OS) excluded patients censored within 1 month from the first administration of nivolumab.

**Results:**

Sixty-seven patients (33.5%) developed thyroid irAEs and these were divided into a subclinical thyroid irAE group (*n* = 40, 20.0%) and an overt thyroid irAE group (*n* = 27, 13.5%). Patients with thyroid uptake of FDG-PET before treatment showed high incidences of overt thyroid irAE (adjusted odds ratio 14.48; 95% confidence interval [CI] 3.12–67.19), while the same relationship was not seen with subclinical thyroid irAE. Regarding the total cohort, the thyroid irAE (+) group had a significantly longer median OS than the thyroid irAE (−) group (16.1 versus 13.6 months, hazard ratio [HR] 0.61; 95% CI 0.39–0.93). In 112 non-excluded patients with lung cancer, the thyroid irAE (+) group similarly had a longer median OS than the thyroid irAE (−) group (not reached versus 14.2 months, HR 0.51; 95% CI 0.27–0.92). However, this observation was not seen in 41 non-excluded patients with malignant melanoma (12.0 versus 18.3 months, HR 1.54; 95% CI 0.67–3.43).

**Conclusions:**

By thyroid uptake of FDG-PET, overt thyroid irAEs could be predicted before nivolumab therapy. Thyroid irAEs related to good prognosis in lung cancer but might be inconclusive in malignant melanoma.

## Introduction

Immune checkpoint inhibitors have been applied to a variety of malignancies [1]. Monoclonal antibodies against cytotoxic T-lymphocyte–associated protein 4 (CTLA-4), programmed cell death-1 (PD-1), and programmed death-ligand 1 have been approved. Immune-related adverse events (irAEs) are commonly caused by these inhibitors and involve several endocrine-related organs. For example, hypophysitis with ipilimumab, an anti-CTLA-4 antibody; and ACTH deficiency, type 1 diabetes, and thyroid dysfunction with nivolumab and pembrolizumab, anti-PD-1 antibodies, have been reported [2].

We previously performed a case-series study of irAEs involving the thyroid gland (thyroid irAEs) induced by nivolumab [3]. Our observation that nivolumab caused a transient and rapid course of thyrotoxicosis and subsequent persistent hypothyroidism seemed to be distinctive compared to conventional painless thyroiditis. Studies regarding pembrolizumab revealed high incidences of thyroid irAEs [4, 5].

From the other perspective, there were several reports that the occurrence of irAEs was related to good prognosis [6–12]. However, a few of those focused on individual irAEs: skin irAEs in malignant melanoma [6], and pneumonia in non-small cell lung cancer (NSCLC) [12]. These two findings might be reasonable because the primary site of the cancer and the organs affected by the irAE were the same. Meanwhile, a curious relationship between thyroid irAEs and good prognosis in NSCLC was reported [7, 9], but further validation is needed because sample sizes of these reports were small (51 [7] and 58 patients [9]).

The present study aimed to gain further understanding of a thyroid irAE using a retrospective cohort of patients who received nivolumab therapy. In particular, we tried to predict thyroid irAE development by parameters available before treatment, and to validate the relationship between thyroid irAEs and good prognosis, including but not limited to lung cancer. If both predictive factors and favorable effects on prognosis of thyroid irAEs are determined, the indication of PD-1 pathway blockade therapy could be optimized.

## Patients and methods

### Patients

A retrospective cohort study was performed using the medical records of patients at Kyoto University Hospital. We selected consecutive patients who were started nivolumab therapy between September 1, 2014 and August 31, 2017, and provided them with opportunity to opt out using our website instead of obtaining informed consent. All data of them were collected and fully anonymized in the secured electronic medical record system, and then the input dataset was transferred and imported to analysis software. This study was approved by the Institutional Review Board and Ethics Committee of the Kyoto University Graduate School of Medicine (approval number, R1400), and was conducted in accordance with the principles of the Declaration of Helsinki.

### Assays

Thyroid function tests which consisted of serum free T3 (fT3), free T4 (fT4), and TSH levels were performed using electrochemiluminescent immunoassay (Elecsys FT3 II kit, Elecsys FT4 kit, and Elecsys TSH kit, respectively; Roche Diagnostics, Mannheim, Germany); the reference ranges were 2.33–4.00 pg/mL, 0.880–1.620 ng/dL, and 0.500–5.000 μIU/mL, respectively. Levels of anti-thyroperoxidase antibodies (TPOAbs) and anti-thyroglobulin antibodies (TgAbs) were also measured using electrochemiluminescent immunoassay (Elecsys Anti-TPO kit and Elecsys Anti-Tg kit, respectively; Roche Diagnostics). Thyroid uptake of FDG-PET was defined as diffusely increased FDG uptake in the thyroid gland.

### Assessments of irAE

Thyroid irAEs were determined by the data of serum fT4 and TSH levels for 6 months from first administration of nivolumab. We excluded patients for which abnormal values for these measurements were not a result of nivolumab therapy according to the following criteria: 1) data of thyroid function tests were unavailable; 2) serum TSH levels were normalized during the follow-up period; 3) total thyroidectomy was previously performed; 4) serum TSH levels did not exceed 10.000 μIU/mL in patients with mild subclinical hypothyroidism (defined as TSH < 10.000 μIU/mL); 5) serum fT4 levels did not fall below the reference range in patients with moderate subclinical hypothyroidism (defined as TSH ≥ 10.000 μIU/mL); and 6) low TSH values were only observed during glucocorticoid therapy. Subsequently, if neither serum fT4 nor TSH levels were normal, patients were classified into an overt thyroid irAE group; others were classified into a subclinical thyroid irAE group.

Non-thyroid irAEs were characterized as adverse events involving organs other than the thyroid gland that required intravenous or oral glucocorticoid therapy for resolution. Best overall responses and progression-free survival (PFS) were determined using the assessment of the physicians based on RECIST (Response Evaluation Criteria in Solid Tumors) version 1.1 [13]. The follow-up periods were at least 6 months and as long as 2 years: patients without death were censored at 2 years since their first administration of nivolumab or at data lock time in February 28, 2018. Seventeen patients were otherwise censored because of hospital transfer or treatment interruption.

### Statistical analysis

The number of treated patients during the study period determined the sample size. It was sufficient to detect an hazard ratio (HR) for overall survival (OS) of 0.45 with 80% power at the 5% level of significance, assuming that incidence of thyroid irAE was approximately 15% [4, 5, 7] and median OS was 40 months in patients without thyroid irAE [7].

Data of continuous variables were expressed as medians (interquartile range). Comparisons of parameters between two groups were analyzed by Mann-Whitney U test or Pearson’s chi-square test, and those among more than two groups were by Kruskal-Wallis test or Pearson’s chi-square test corrected by Bonferroni’s method. Odds ratios were calculated and adjusted by logistic regression model. Landmark cohorts were used for ad hoc analyses of OS in lung cancer subgroup [14]. OS and PFS were expressed by the Kaplan-Meier method, and comparisons of them were analyzed by Cox proportional hazards model between two groups and by generalized Wilcoxon test corrected by Bonferroni’s method among three groups. Cox proportional hazards model was also used for multivariate analyses of OS. JMP pro version 11.3.0 (SAS institute Inc., Cary, NC) was used for all statistical analyses. A *P*-value of < 0.05 was considered statistically significant.

## Results

### Incidence and classification of thyroid irAE

Characteristics of the 200 recruited patients are presented in S1 Table. Prior chemotherapy had been performed in all 118 patients with lung cancer, but only in 16 of 42 patients (38.1%) with malignant melanoma. No patients had received prior immune checkpoint blockade therapies. The patients received nivolumab at 2 mg/kg every 3 weeks (for malignant melanoma) or 3 mg/kg every 2 weeks (for lung cancer and other malignancies). The median duration of nivolumab therapy was 69 (28–163) days. Thyroid function were tested every 3 weeks in patients with malignant melanoma and every 4 weeks in patients with lung cancer and other malignancies during nivolumab therapy as a general rule in Kyoto University Hospital. The compliance rates of thyroid function tests were 88.6%, 93.8%, and 90.1% in malignant melanoma, lung cancer, and other malignancies, respectively. TPOAbs and TgAbs were tested in 17 patients (8.5%) and FDG-PET scans were performed in 111 patients (55.5%). Other parameters were available from all patients.

**Fig 1:**
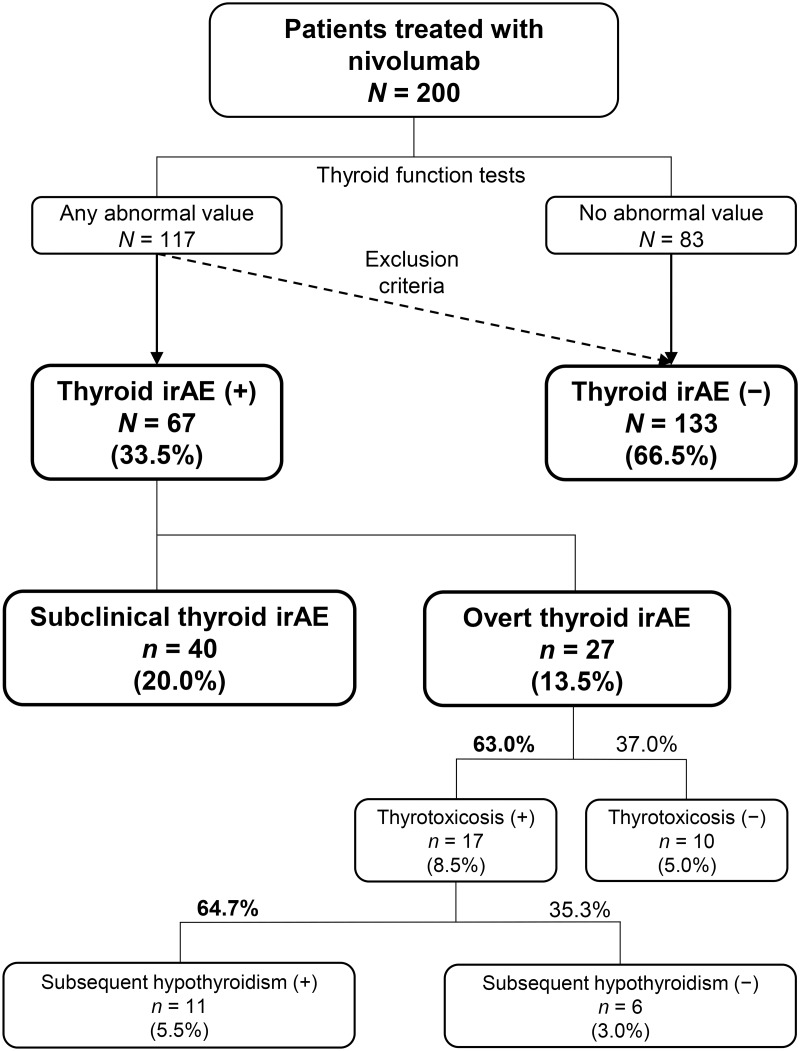
A flowchart for determination of immune-related adverse events involving the thyroid gland (thyroid irAEs). Determination and classifications were based on data of thyroid function tests.

We checked all of the data of the thyroid function tests of these patients and systematically classified them as shown in Fig 1. The thyroid irAE (+) group (*n* = 67, 33.5%) was divided into the subclinical thyroid irAE group (*n* = 40, 20.0%) and the overt thyroid irAE group (*n* = 27, 13.5%).

The subclinical thyroid irAE group consisted of 22 patients with normal fT4 and high TSH levels, 7 with high fT4 and normal TSH, 6 with low fT4 and normal TSH, and 5 with normal fT4 and low TSH. Of the 6 patients with low fT4 and normal TSH levels, 4 patients were accompanied by marked decreases in fT3 levels (1.01, 1.32, 1.41, and 1.54 pg/mL), and other 2 patients only showed slight low fT4 levels (0.878 and 0.867 ng/dL). These did not suggest development of central hypothyroidism due to hypophysitis as non-thyroid irAEs. Of the 27 patients with overt thyroid irAEs, transient thyrotoxicosis was observed in 17 patients (63.0%); 11 of these 17 patients (64.7%) developed subsequent hypothyroidism.

High fT3 values were observed in 18 patients; 13 patients also presented high fT4 values and other 5 patients did not present any abnormal TSH values. High TSH values were observed in 48 of 125 patients (38.4%) with low fT3 values and in 24 of 36 patients (66.7%) with low fT4 values. Thus, fT4-based determination was more specific both for thyrotoxicosis and hypothyroidism.

### Prediction of thyroid irAE

We performed subgroup analyses of the subclinical thyroid irAE group and the overt thyroid irAE group compared to the thyroid irAE (−) group (Table 1 and S2 Table). Of parameters available before nivolumab therapy, the overt thyroid irAE group showed significant differences in sex, thyroid uptake of FDG-PET, and serum TSH levels (Table 1). In multiple logistic regression model with these three parameters, thyroid uptake of FDG-PET was only associated with overt thyroid irAE development (Adjusted odds ratio [OR] 14.48; 95% confidence interval [CI] 3.12–67.19, *P* < 0.001) (Table 2). There were no significant differences in plasma glucose levels at FDG administration (*P* = 0.540): the medians were 115 mg/dL (106–132), 119 mg/dL (107–141), and 111 mg/dL (101–121) in the thyroid irAE (−) group, the subclinical thyroid irAE group, and the overt thyroid irAE group, respectively.

**Table 1:** Characteristics of patients with subclinical and overt thyroid irAEs and comparisons to those without thyroid irAEs.

	Thyroid irAE (−)	Thyroid irAE (+)			
	n = 133	Subclinical	p	Overt	p
		n = 40		n = 27	
Age (years)	67 (60–73)	69 (58–75)	0.701	67 (60–73)	0.958
Gender (n)			0.420		**0.022**
Male	94 (70.7%)	27 (67.5%)		13 (48.2%)	
Female	39 (29.3%)	13 (32.5%)		14 (51.8%)	
Primary sites (n)			0.286		0.409
Lung cancer	77 (57.9%)	29 (72.5%)		12 (44.5%)	
Malignant melanoma	28 (21.05%)	6 (15.0%)		8 (29.6%)	
Others	28 (21.05%)	5 (12.5%)		7 (25.9%)	
**Results before nivolumab therapy**					
Positive TPOAb (n)	0 (tested in 7) (0.0%)	1 (tested in 4) (25.0%)	NA	3 (tested in 6) (50.0%)	NA
Positive TgAb (n)	0 (tested in 7) (0.0%)	1 (tested in 4) (25.0%)	NA	5 (tested in 6) (83.3%)	NA
Thyroid uptake of FDG-PET (n)	3 (tested in 71) (4.2%)	1 (tested in 25) (4.0%)	1.000	7 (tested in 15) (46.7%)	**< 0.001**
Thyroid function					
free T3 (pg/mL)	2.57 (2.20–2.89)	2.49 (2.25–2.83)	0.684	2.59 (2.18–2.82)	0.895
free T4 (ng/dL)	1.250 (1.100–1.380)	1.270 (1.100–1.360)	0.979	1.160 (0.946–1.350)	0.184
TSH (μIU/mL)	2.260 (1.353–4.443)	2.580 (0.935–4.430)	0.940	3.620 (1.870–7.310)	**0.014**

Data of continuous variable were expressed as medians (interquartile range). Statistical analyses were performed against thyroid irAE (–) group.

We did not perform statistical analysis of TPOAb and TgAb results because the numbers of missing values were large.

n: number of subjects, irAE: immune-related adverse event, NA: not available

**Table 2:** Odds ratio for overt thyroid irAE development.

	OR/Adjusted OR (95% CI)	p
**Univariate analysis**		
Sex (Female > Male)	2.60 (0.86–7.85)	0.090
Thyroid uptake of FDG-PET	19.91 (4.79–82.79)	**< 0.001**
TSH > 2.5 μIU/mL	3.06 (0.91–10.28)	0.071
**Multivariate analysis**		
Sex (Female > Male)	1.62 (0.41–6.37)	0.493
Thyroid uptake of FDG-PET	14.48 (3.12–67.19)	**< 0.001**
TSH > 2.5 μIU/mL	2.68 (0.67–10.67)	0.162

Statistical analysis was performed using logistic regression model.

Results of thyroid uptake of FDG-PET and TSH were obtained before nivolumab therapy.

OR: odds ratio, CI: confidence interval

### Clinical features of overt thyroid irAE

Detailed clinical data of 27 patients with overt thyroid irAEs are presented in S3 Table (17 patients with thyrotoxicosis) and S4 Table (10 patients without thyrotoxicosis). In 17 patients, TPOAbs and TgAbs were tested at the point of thyroid dysfunction development; seven patients were double positive, 5 were double negative, and 5 were exclusively positive for TgAbs, while no patients were exclusively positive for TPOAbs.

**Fig 2:**
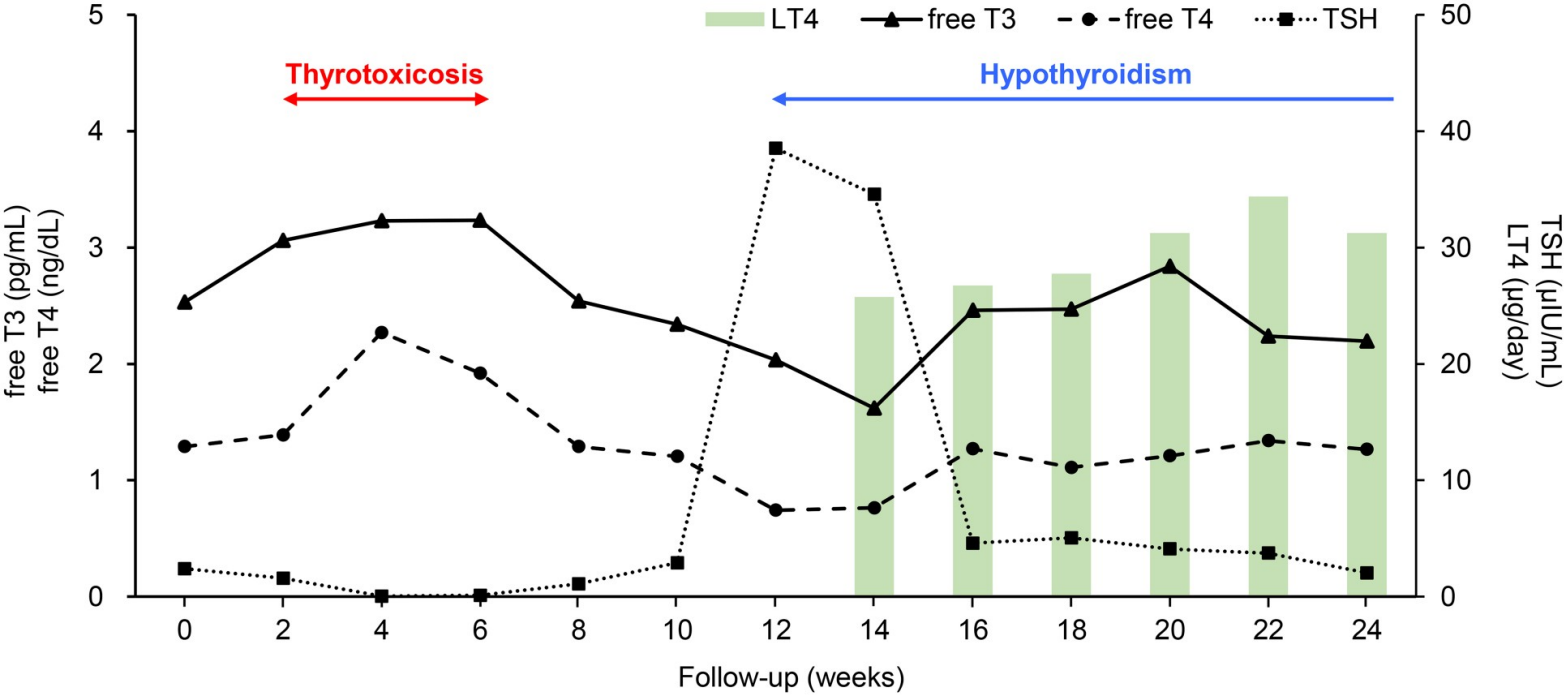
The representative clinical course of overt thyroid irAE. Regarding 17 patients with overt thyroid irAEs and thyrotoxicosis, the medians of the results of thyroid function tests and doses of levothyroxine replacement were plotted. For clarity, the values of 3, 9, 15, and 21 weeks in patients with malignant melanoma were integrated into those of 4, 10, 16, and 22 weeks, respectively.

Thyrotoxicosis frequently developed within 4 weeks from the first administration of nivolumab; 4 patients developed thyrotoxicosis at 2 weeks, 3 at 3 weeks, and 3 at 4 weeks. Subsequent hypothyroidism was more frequently observed in patients that developed thyrotoxicosis within 4 weeks (8 of 10 patients, 80%) than in patients that developed thyrotoxicosis after 4 weeks (3 of 7 patients, 43%). We plotted all of the data (S1 Fig) and the medians (Fig 2) of the thyroid function tests and levothyroxine doses of the 17 patients with thyrotoxicosis to understand the time course. Thyrotoxicosis tended to occur transiently from 2 to 6 weeks, and subsequent hypothyroidism tended to develop from 12 weeks; then, persistent levothyroxine replacement had been performed.

Furthermore, relevance of thyroid uptake of FDG-PET to the clinical features of thyroid irAE was investigated. We could obtain simultaneous results of 7 patients on TPOAb, TgAb, and FDG-PET by including data after the first administration of nivolumab (S3 and S4 Tables), and find the 6 patients with thyroid uptake of FDG-PET were all positive for TgAb (Case No: T4, T6, T8, T11, T17, and H2). Meanwhile, 6 of 7 patients who presented the maximum standardized uptake value (SUVmax) ≥ 4.0 at thyroid developed severe hypothyroidism (defined as TSH > 100 μIU/mL) (Case No: T4, T6, T11, T17, H2, H3, and H7).

### Prognosis of patients with thyroid irAEs

To prevent immortal time bias, we excluded patients censored within 1 month from the first administration of nivolumab for OS analyses, as the early onset was the representative course of thyroid irAE. The results of cohorts including all patients are shown in S2 Fig. The numbers of excluded patients with deaths were 9, 6, and 1, and those with progressive diseases were 25, 15, and 5 in the total cohort, the lung cancer subgroup, and the malignant melanoma subgroup, respectively.

**Fig 3:**
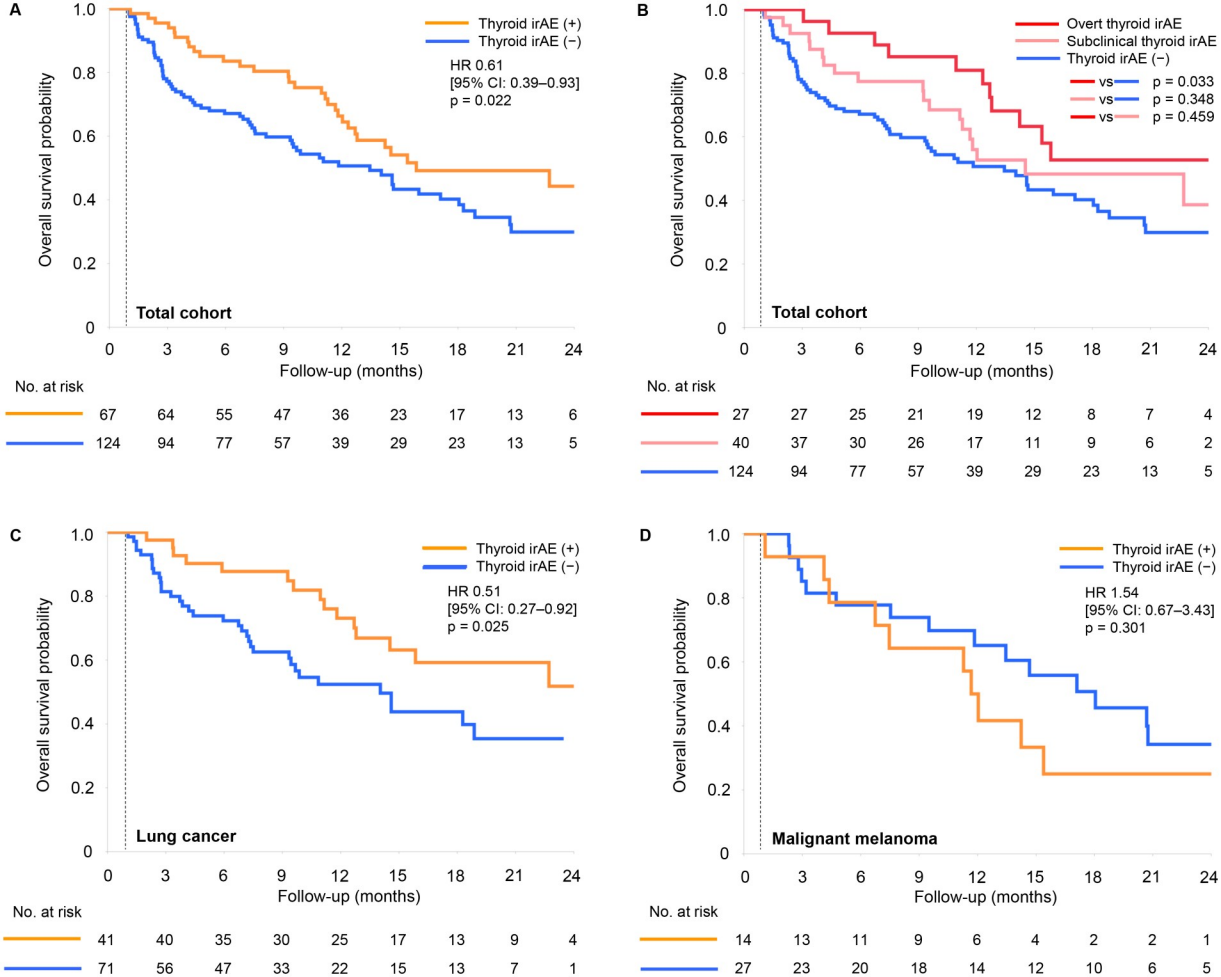
Kaplan-Meier curves of overall survival according to thyroid irAEs. Patients censored within 1 month from the first administration of nivolumab were excluded from each cohort. (A) Results in the total cohort; (B) results of comparisons between the overt thyroid irAE group and the subclinical thyroid irAE group in the total cohort; (C) results in the cohort of lung cancer; (D) results in the cohort of malignant melanoma. HR, hazard ratio; CI, confidence interval.

We observed that the median OS was significantly longer in the thyroid irAE (+) group than in the thyroid irAE (−) group (16.1 versus 13.6 months, hazard ratio [HR] 0.61; 95% CI 0.39–0.93, *P* = 0.022, Fig 3A). When the total cohort divided according to overt and subclinical thyroid irAE, the median OS was significantly longer in the overt thyroid irAE group than in the thyroid irAE (−) group (*P* = 0.033, Fig 3B), but the subclinical thyroid irAE group was not (*P* = 0.348, Fig 3B). There was no difference between the overt and the subclinical thyroid irAE groups (*P* = 0.459, Fig 3B).

An analysis of 112 non-excluded patients with lung cancer showed similar results; the median OS was significantly longer in the thyroid irAE (+) group than in the thyroid irAE (−) group (not reached versus 14.2 months, HR 0.51; 95% CI 0.27–0.92, *P* = 0.025, Fig 3C). However, in 41 non-excluded patients with malignant melanoma, this observation was not seen (12.0 versus 18.3 months in the thyroid irAE (+) group and the thyroid irAE (−) group, respectively, HR 1.54; 95% CI 0.67–3.43, *P* = 0.301, Fig 3D). In Cox proportional hazards models in lung cancer, there were no significant differences in prior radiation therapy, histology (adenocarcinoma or squamous cell carcinoma), and prior epidermal growth factor receptor-tyrosine kinase inhibitor therapy (S5 Table).

**Fig 4:**
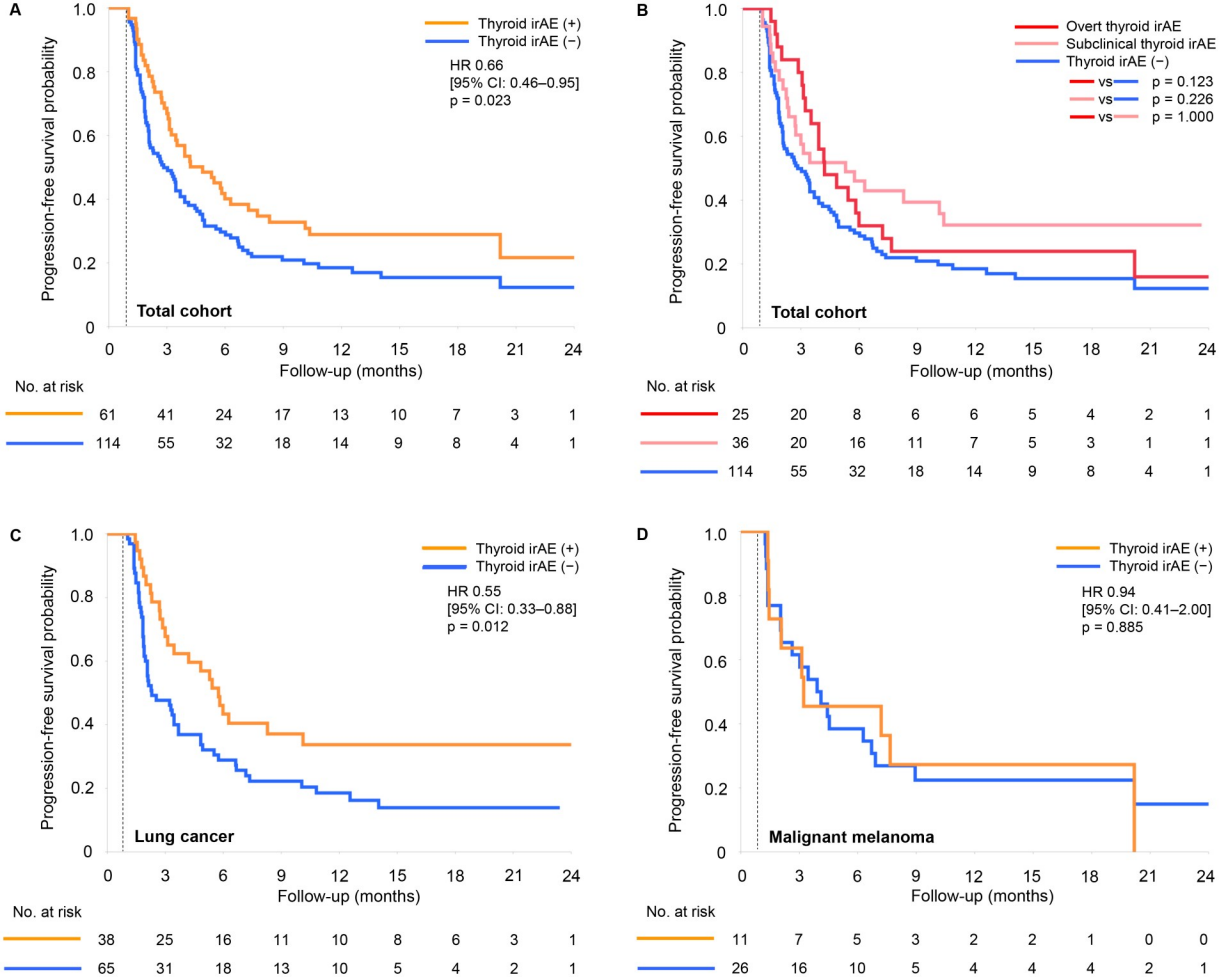
Kaplan-Meier curves of progression-free survival according to thyroid irAEs. Patients censored within 1 month from the first administration of nivolumab were excluded from each cohort. (A) Results in the total cohort; (B) results of comparisons between the overt thyroid irAE group and the subclinical thyroid irAE group in the total cohort; (C) results in the cohort of lung cancer; (D) results in the cohort of malignant melanoma.

PFS was analyzed in a similar manner. The median PFS was significantly longer in the thyroid irAE (+) group than in the thyroid irAE (−) group (4.9 versus 2.9 months, HR 0.66; 95% CI 0.46–0.95, *P* = 0.023, Fig 4A). There were no significant differences in either of the overt and the subclinical thyroid irAE group, compared to the thyroid irAE (−) group (*P* = 0.123 and 0.226, respectively, Fig 4B). In 103 non-excluded patients with lung cancer, the median PFS was significantly longer in the thyroid irAE (+) group than in the thyroid irAE (−) group (5.8 versus 2.3 months, HR 0.55 95% CI 0.33–0.88, *P* = 0.012, Fig 4C), but in 37 non-excluded patients with malignant melanoma, no significant difference was seen as well as OS (3.3 versus 4.1 months in the thyroid irAE (+) group and the thyroid irAE (−) group, respectively, HR 0.94; 95% CI 0.41–2.00, *P* = 0.885, Fig 4D).

Thyroid irAE seemed to relate to good prognosis in lung cancer, but immortal time bias still remained because we determined thyroid irAEs by the data for 6 months from first administration of nivolumab. We planned ad hoc landmark analyses regarding OS of the lung cancer subgroup: censored patients were excluded and thyroid irAEs were determined by data up to the landmarks of 1 month, 2 months, 3 months, and 6 months. Statistical power was calculated at the 5% level of significance to detect effect size of 30%: this calculation was based on the results regarding Fig 3 that OS probabilities at 12 months in the lung cancer cohort were 71.4% (n = 35) and 42.3% (n = 52) in the thyroid irAE (+) group and the thyroid irAE (−) group, respectively. In 1-month landmark analysis, the power was 94.9%, assuming 12 patients developed thyroid irAE within 1 month and 75 patients did not, as the case series of overt thyroid irAE (S3 and S4 Tables): in 6-month landmark analysis, the power was 94.8%, assuming 26 patients developed thyroid irAE and 39 patients did not, as 29 of 112 patients (25.9%) were intended to be excluded. Thus, the ad hoc analyses had sufficient power.

Kaplan-Meier curves of OS in the ad hoc landmark analyses are shown in S3 Fig. We compared OS probability at 12 months after the first administration of nivolumab because Cox proportional hazards model was not applicable in 1-month, 2-month, and 3-month landmark cohorts. The thyroid irAE (+) group showed significant higher OS probability than the thyroid irAE (−) group in the 1-month landmark analysis (76.5% versus 48.6%, P = 0.038) and the 2-month landmark analysis (73.1% versus 50.0%, P = 0.049). Significant changes were not seen in the 3-month landmark analysis (75.0% versus 57.8%, P = 0.135) and the 6-month landmark analysis (83.3% versus 64.7%, P = 0.092). Meanwhile, we verified thyroid uptake of FDG-PET was significantly associated with overt thyroid irAE development within 1 month, 2 months, and 3 months (S6 Table) as well as 6 months (Table 1).

To verify whether this discrepancy was limited to thyroid irAEs, we analyzed 4 subgroups; thyroid irAE(−) and non-thyroid irAE(−) as a no irAE group (n = 47 in lung cancer, n = 18 in malignant melanoma); thyroid irAE(+) and non-thyroid irAE(−) as a thyroid irAE group (n = 27, n = 10); thyroid irAE(−) and non-thyroid irAE(+) as a non-thyroid irAE group (n = 5, n = 5); and thyroid irAE(+) and non-thyroid irAE(+) as a both irAE group (n = 8, n = 3). Detailed characteristics are presented in S7 Table and Kaplan-Meier curves of OS probability are in S4 Fig. In the lung cancer subgroup, the thyroid irAE group showed significant higher OS probability at 12 months after the first administration of nivolumab than no irAE group (70.4% versus 40.4%, *P* = 0.013), while the non-thyroid irAE group did not (60.0% vs 40.4%, *P* = 0.400). Conversely, in the malignant melanoma subgroup, the non-thyroid irAE group showed significant higher OS probability (100.0% vs 50.0%, *P* = 0.043), while the thyroid irAE group did not (50.0% vs 50.0%, *P* = 1.000).

## Discussion

We conducted this retrospective cohort study to obtain clinically useful findings of thyroid irAEs. Overt thyroid irAEs frequently developed in patients positive for thyroid uptake of FDG-PET, while subclinical thyroid irAEs did not. In representative patients with overt thyroid irAEs, transient thyrotoxicosis promptly occurred within 2 to 6 weeks after the first administration of nivolumab, and subsequent hypothyroidism requiring persistent levothyroxine replacement from 12 weeks. The finding with the most clinical influence was the relationship between thyroid irAEs and good prognosis. However, this relationship was significant and independent of prior therapy in the lung cancer subgroup, but not significant in the malignant melanoma subgroup.

The present study provided the first data of a large cohort regarding nivolumab-induced thyroid irAE. In addition, we could evaluate exclusive effects of PD-1 pathway blockade therapy because our cohort did not include any patients who had received prior immune checkpoint blockade therapies such as ipilimumab, unlike two previous studies [4, 5]. Our results showed a higher incidence of thyroid irAE than previous reports regarding pembrolizumab; 17 of 99 patients (17.2%) [4], 13 of 93 patients (14.0%) [5], and 10 of 48 patients (20.8%) [7]. This might have been caused by our systematic determination that could even detect subclinical thyroid irAEs, as the incidence of overt thyroid irAE was similar to the above reports [4, 5]. Likewise, the incidence of thyroid irAE in the present study was higher than that of a meta-analysis of clinical trials of anti-PD-1 and anti-PD-L1 antibodies; hypothyroidism was 6.07% and thyrotoxicosis was 2.82% [15]. This discrepancy between a real-world setting and clinical trials suggested that the difference of definitions and screening methods was a matter to be considered. On the other hand, we confirmed that the most frequent course of thyroid irAE is a transient thyrotoxicosis with subsequent hypothyroidism, as seen in our previous case series [3] and the previous report [4].

It was reported that TPOAbs and TgAbs were often positive in patients with thyroid irAEs [9, 16, 17]. Based on a report that only positive results for TPOAbs were significantly associated with hypothyroidism [18], the TgAb test has not been recommended for management of hypothyroidism [19]. However, as there were 5 patients exclusively positive for TgAb and no patients exclusively positive for TPOAb in our case series, TgAb rather than TPOAb seemed to be related to overt thyroid irAEs. The number of examined patients was small, so further prospective studies are needed. There was another interesting observation that 5 patients were double negative for TPOAbs and TgAbs, which provided a hypothesis that thyroid irAEs are not always caused by these antibodies, but by antibodies for unknown antigens or unknown mechanisms unrelated to humoral immunity.

It is known that thyroid uptake of FDG-PET could be positive at the period of thyroid irAE development [4, 16]. We additionally confirmed that thyroid uptake of FDG-PET before treatment increased the risk of overt thyroid irAE development. It also needs to be noted that relative high thyroid uptake of FDG-PET, defined as SUVmax ≥ 4.0 in the present study, could suggest development of severe subsequent hypothyroidism. If high thyroid uptake of FDG-PET is seen, we might have to follow more closely to diagnose and treat hypothyroidism without delay.

From other perspective, all examinable patients with thyroid uptake of FDG-PET were positive for TgAb, but positive results of TPOAb were limited to a part of them. Thyroid uptake of FDG-PET has been considered to be a result of thyroiditis, while it is not associated with TPOAb levels [20]. The present observation also supports the notion that the significance of TgAb in thyroid irAEs is worth examining.

In addition, thyroid uptake of FDG-PET, as a predictive factor of thyroid irAEs that is available before treatment, has become important because thyroid irAEs have been revealed to be a prognostic factor. Our landmark analyses using the larger cohort provided further evidences of this interesting relationship that was previously suggested by two previous reports that non-landmark analyses were performed using the smaller cohorts [7, 9]. At this point, the multiple landmark analyses supported that thyroid irAE development within 2 months had relevant association with good prognosis. However, further careful examinations are needed for confirmation because these analyses were just ad hoc. Overt thyroid irAEs are thought to be more meaningful because they are predictable by thyroid uptake of FDG-PET and their relationships to prolonged OS and PFS have been confirmed. Subclinical thyroid irAEs are needed for further examinations as they have not been established as a prognostic factor and their prediction by surveillance before treatment is unable at this point.

Our analyses involving multiple primary sites revealed a very important observation; thyroid irAEs were a possible prognostic factor for lung cancer, but might not for malignant melanoma. It should be noted that the evidences on thyroid irAE from previous reports [7, 9] and the present study could be applied only to lung cancer. Although the number of patients was small, non-thyroid irAEs significantly related to good prognosis in malignant melanoma. There remains a possibility that thyroid irAEs has the weak relationship with prognosis compared to non-thyroid irAEs in malignant melanoma. Other malignancies certainly need individual examinations.

We suggest a hypothesis that target antigens common to the lung and the thyroid gland exist, to explain this crucial finding. The evidence that irAEs are likely to develop in the same organs with primary sites might support this hypothesis: pneumonia is common in lung cancer, while skin irAEs are common in malignant melanoma [6]. If thyroid irAEs are caused by antibodies recognizing antigens common to the lung, immune responses to lung cancer are expected.

There were several limitations due to the retrospective design. The number of patients tested with TPOAbs and TgAbs was small, and stages of malignancies and their treatments prior to or post nivolumab were heterogeneous. However, evidence in this field is still limited because difficulties in collecting prospective data have arisen due to the rarity of thyroid irAEs and ethical considerations. Our findings could contribute to clinical settings and provide novel hypotheses.

In conclusion, overt thyroid irAEs have a stronger impact than subclinical thyroid irAEs because they could be predicted before treatment by thyroid uptake of FDG-PET. Development of thyroid irAEs related to good prognosis in the total cohort and the lung cancer subgroup, but might not in the malignant melanoma subgroup. Considering the predictive factors of overt thyroid irAEs could be a promising strategy for PD-1 pathway blockade therapy.

## Supporting information

S1 Fig Individual results of 17 patients with thyrotoxicosis. (A)(B)(C) Line graphs of results of thyroid function tests of 17 patients with overt immune-related adverse events involving the thyroid gland and thyrotoxicosis. (D) A box plot of doses of levothyroxine (LT4) replacement for these 17 patients. Each box represents the interquartile range. The horizontal line in each box represents the median. The ends of the vertical lines represent the minimum and maximum values.
(TIF)

S2 Fig Kaplan-Meier curves of overall survival according to thyroid irAEs in cohorts including all patients. (A) Results in the total cohort; (B) results of comparisons between the overt thyroid irAE group and the subclinical thyroid irAE group in the total cohort; (C) results in the lung cancer subgroup; (D) results in the malignant melanoma subgroup. HR, hazard ratio; CI, confidence interval.
(TIF)

S3 Fig Kaplan-Meier curves of overall survival in ad hoc landmark analyses of lung cancer. Thyroid irAEs were determined by data up to each landmark and patients censored within the landmarks were excluded. (A) Results in the 1-month landmark cohort, (B) results in the 2-month landmark cohort, (C) results in the 3-month landmark cohort, and (D) results in the 6-month landmark cohort. Statistical analyses were performed for overall survival probabilities at 12 months after the first administration of nivolumab. irAE, immune-related adverse event.
(TIF)

S4 Fig Kaplan-Meier curves of overall survival according to thyroid irAEs and non-thyroid irAEs. Patients censored within 1 month from the first administration of nivolumab were excluded from each cohort. (A) Results in the cohort of lung cancer; (B) results in the cohort of malignant melanoma. Statistical analyses were performed for overall survival probabilities at 12 months after the first administration of nivolumab against no irAE group. irAE, immune-related adverse event.
(TIF)

S1 Table Patient characteristics of a total cohort and subgroups according to primary sites. (XLSX)

S2 Table Additional characteristics of patients with subclinical and overt thyroid irAEs. (XLSX)

S3 Table Detailed clinical data of patients with overt thyroid irAEs who developed thyrotoxicosis. (XLSX)

S4 Table Detailed clinical data of patients with overt thyroid irAEs who did not develop thyrotoxicosis. (XLSX)

S5 Table Cox proportional hazards models of overall survival in lung cancer. (XLSX)

S6 Table Characteristics of patients with thyroid irAEs and comparisons to those without thyroid irAEs in ad hoc landmark analyses. (XLSX)

S7 Table Patient characteristics of subgroups according to thyroid irAEs and non-thyroid irAEs. (XLSX)
