# Supplementary figures and images for: Incidence, features, and prognosis of immune-related adverse events involving the thyroid gland induced by nivolumab
Source: PLoS One. 2019 May 14;14(5):e0216954. doi: 10.1371/journal.pone.0216954 (PMC6516638; doi:10.1371/journal.pone.0216954)

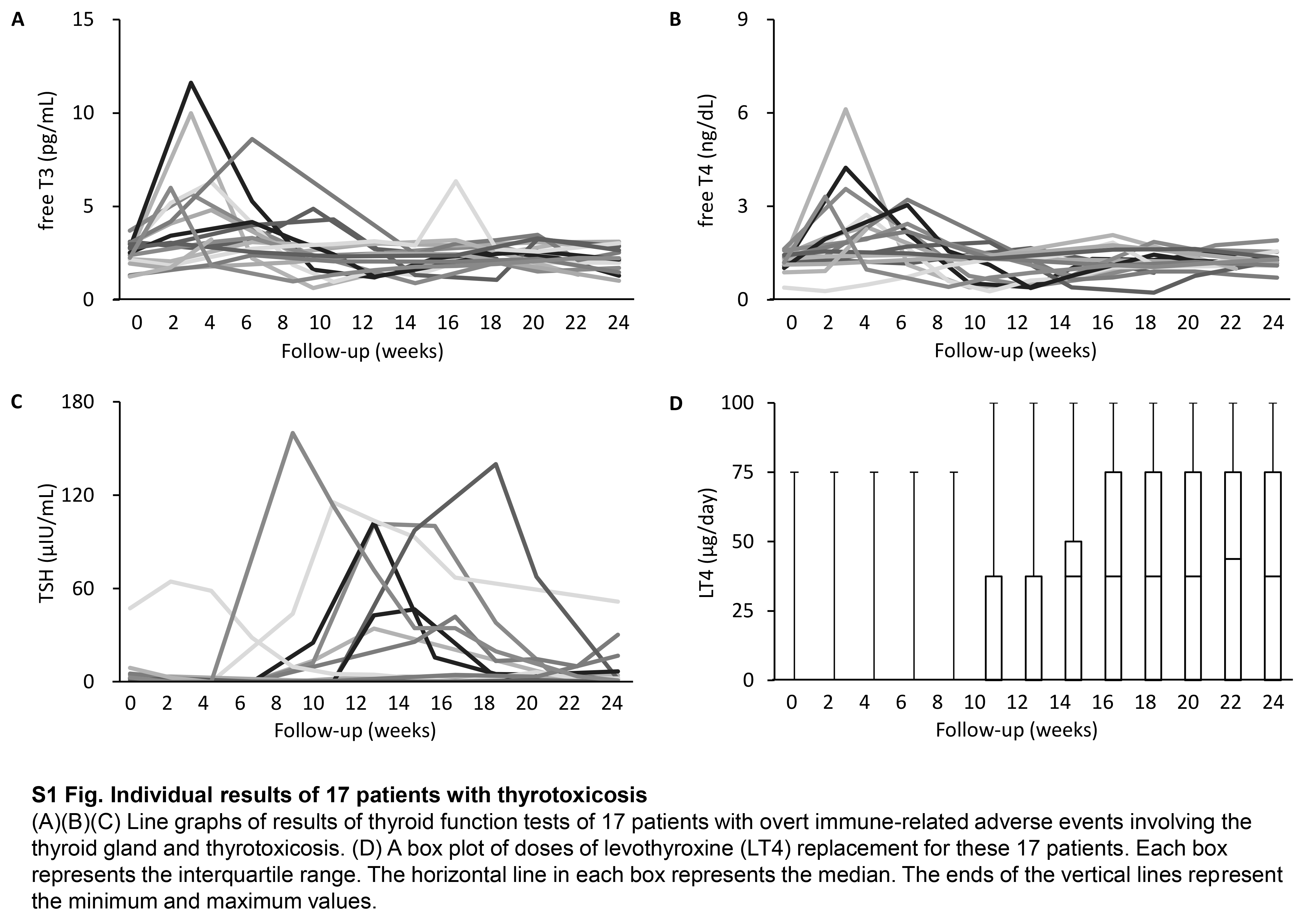

Supplement: S1 Fig — (A)(B)(C) Line graphs of results of thyroid function tests of 17 patients with overt immune-related adverse events involving the thyroid gland and thyrotoxicosis. (D) A box plot of doses of levothyroxine (LT4) replacement for these 17 patients. Each box represents the interquartile range. The horizontal line in each box represents the median. The ends of the vertical lines represent the minimum and maximum values. (TIF) [file pone.0216954.s001.tif]

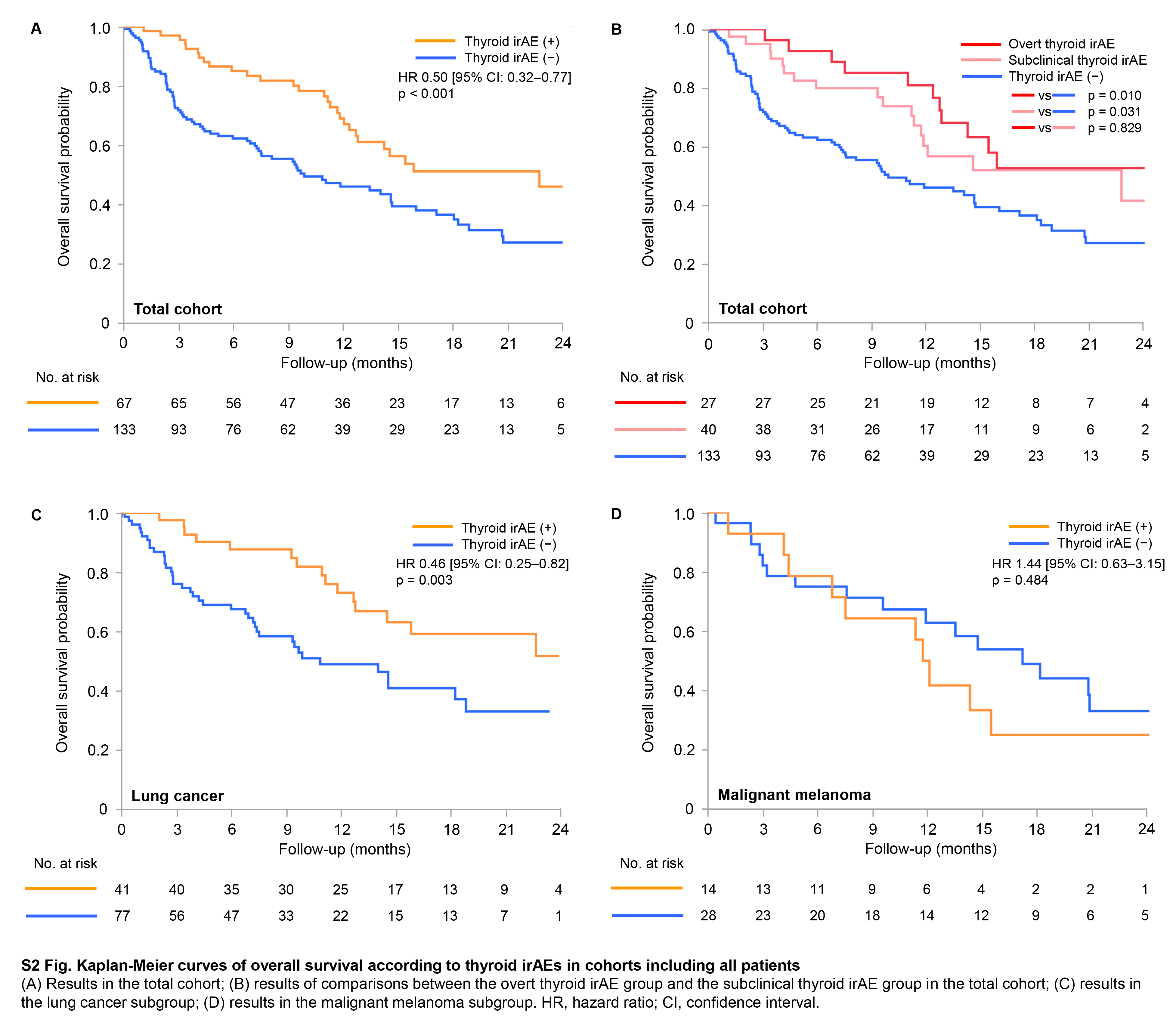

Supplement: S2 Fig — (A) Results in the total cohort; (B) results of comparisons between the overt thyroid irAE group and the subclinical thyroid irAE group in the total cohort; (C) results in the lung cancer subgroup; (D) results in the malignant melanoma subgroup. HR, hazard ratio; CI, confidence interval. (TIF) [file pone.0216954.s002.tif]

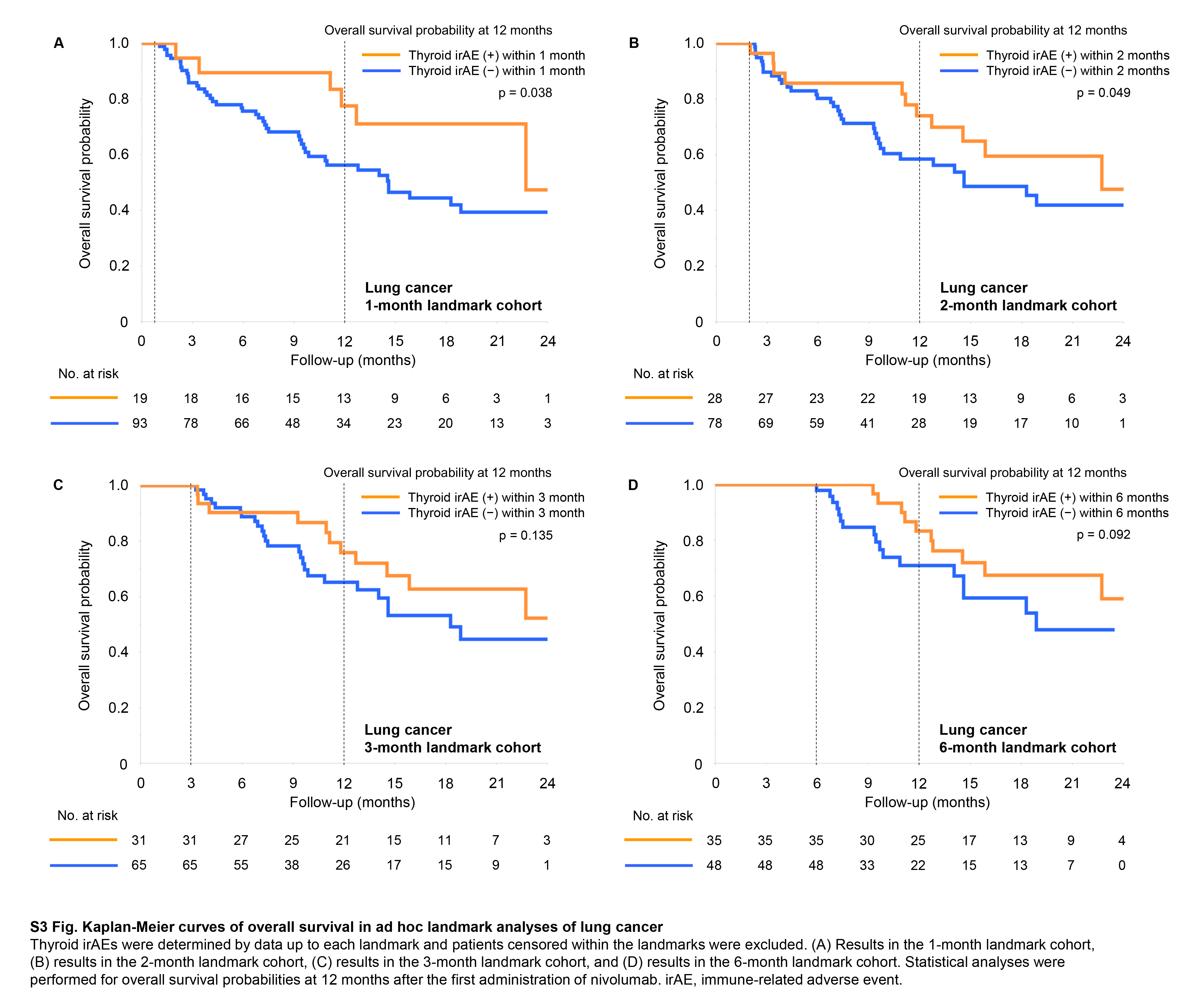

Supplement: S3 Fig — Thyroid irAEs were determined by data up to each landmark and patients censored within the landmarks were excluded. (A) Results in the 1-month landmark cohort, (B) results in the 2-month landmark cohort, (C) results in the 3-month landmark cohort, and (D) results in the 6-month landmark cohort. Statistical analyses were performed for overall survival probabilities at 12 months after the first administration of nivolumab. irAE, immune-related adverse event. (TIF) [file pone.0216954.s003.tif]

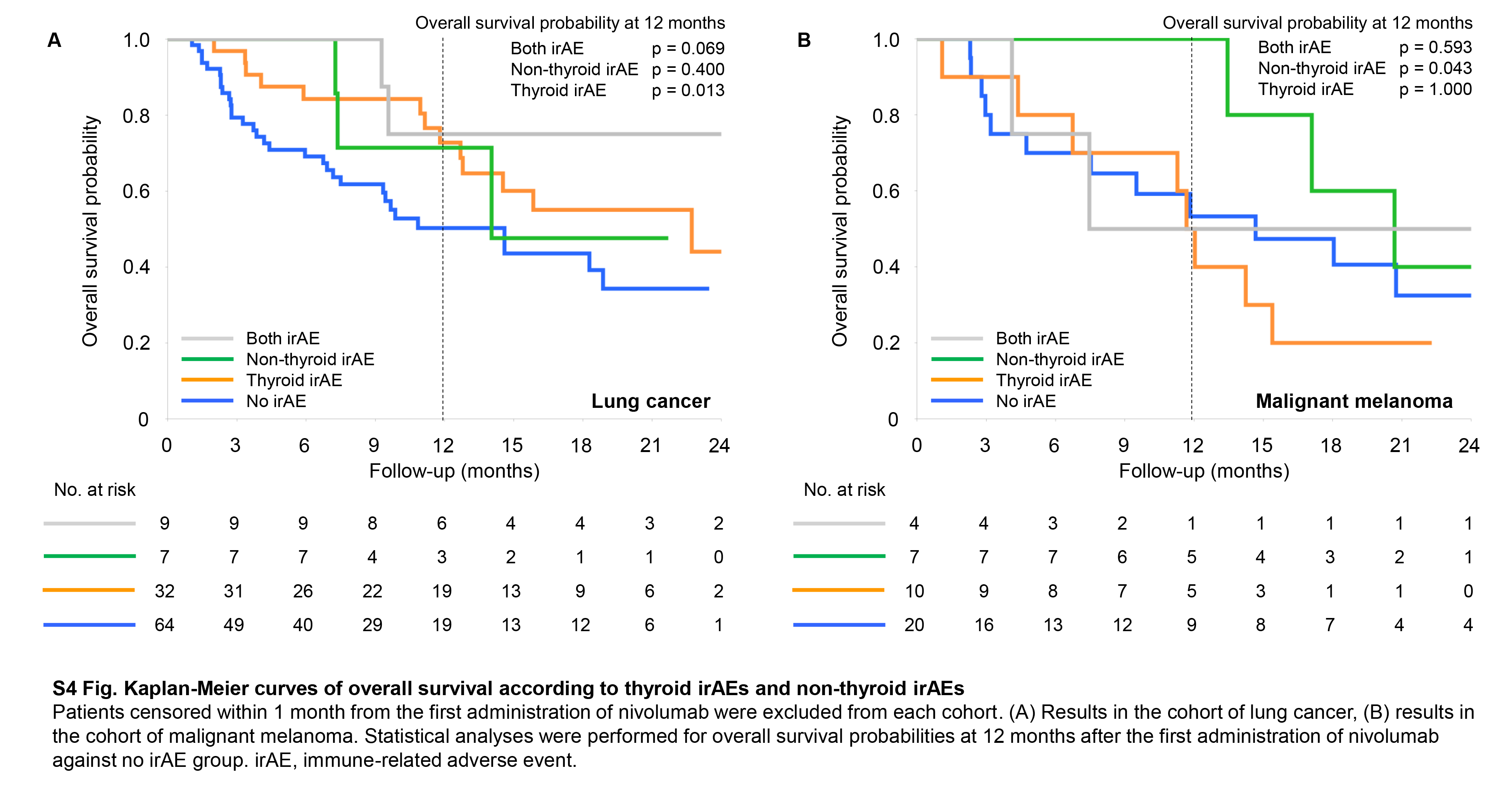

Supplement: S4 Fig — Patients censored within 1 month from the first administration of nivolumab were excluded from each cohort. (A) Results in the cohort of lung cancer; (B) results in the cohort of malignant melanoma. Statistical analyses were performed for overall survival probabilities at 12 months after the first administration of nivolumab against no irAE group. irAE, immune-related adverse event. (TIF) [file pone.0216954.s004.tif]
